# The Role of Individually Selected Diets in Obese Women with PCOS—A Review

**DOI:** 10.3390/nu14214555

**Published:** 2022-10-28

**Authors:** Izabela Chudzicka-Strugała, Iwona Gołębiewska, Beata Banaszewska, Grzegorz Brudecki, Barbara Zwoździak

**Affiliations:** 1 Department of Medical Microbiology, Poznan University of Medical Sciences, Wieniawskiego 3, 61-712 Poznan, Poland; 2 Earth and Life Institute (ELI), UCLouvain, Croix du Sud 2, 1348 Louvain-La-Neuve, Belgium; 3 Chair and Department of Laboratory Diagnostics, Poznan University of Medical Sciences, 60-533 Poznan, Poland; 4 Group 42 (Healthcare), Masdar City, Abu Dhabi P.O. Box 112778, United Arab Emirates

**Keywords:** polycystic ovary syndrome, obesity/overweight, diet, probiotics/synbiotic

## Abstract

Polycystic ovarian syndrome (PCOS) is one of the most common heterogeneous endocrine and metabolic disorders in premenopausal women. It is a complex multifactorial disorder with strong epigenetic and environmental influences, including factors related to eating habits and lifestyle. There is a close relationship between obesity and PCOS. Weight gain and obesity are often clinical symptoms manifested by biochemical markers. Moreover, abdominal obesity in women with PCOS is involved in the development of inflammatory changes. A significant share of balanced therapies correcting the lifestyle of patients is suggested, e.g., with the implementation of appropriate diets to minimize exposure to inflammatory factors and prevent abnormal immune system stimulation. In the case of obese patients with PCOS, planning a diet program and supporting the motivation to change eating habits play an important role to lose weight and lower BMI. Probiotics/synbiotic supplementation may enhance weight loss during the diet program and additionally positively affect metabolic and inflammatory factors by improving the intestinal microbiome.

## 1. Introduction

Polycystic ovarian syndrome (PCOS) is one of the most common heterogeneous endocrine and metabolic disorders in premenopausal women. The etiology of this disease has not been fully understood so far, and its pathogenesis is suggested primarily by genetic factors, patients’ lifestyles, and insulin resistance. Many scientific studies suggest that PCOS is a complex multifactorial disorder with strong epigenetic and environmental influences, including factors related to eating habits and lifestyle [1]. The incidence of this endocrinopathy in women of reproductive age ranges from 5% to 10%, and sometimes is even at the level of 26%, depending on the included diagnostic criteria and the population studied [1,2].

Polycystic ovary syndrome can manifest itself at any stage of reproductive life, but it is often related to adolescence. PCOS is characterized by reproductive and hyperandrogenic features, which include oligo- or amenorrhea, impaired fertility, hirsutism, acne, and androgenic alopecia, as well as concomitant hyperandrogenemia. PCOS is an obesity-related disorder, thus weight gain and obesity contribute to the development of PCOS syndrome. There are mechanisms by which the development of PCOS may contribute to further weight gain and hinder effective weight loss. Hence, there is a close relationship between obesity and PCOS. Additionally, in genetically predisposed women, weight gain and obesity are important risk factors for the clinical and biochemical manifestations of PCOS [3].

Based on the latest literature, obese women with PCOS (BMI > 30) showed an almost 4 times higher risk of IGT (impaired glucose tolerance) or T2DM (Type 2 Diabetes Mellitus) than normal glucose tolerance (NGT), and the high incidence of obesity in PCOS further increases the risk of IR and T2DM. Therefore, it is suggested that there is a relationship between the incidence of obesity in PCOS and the increased risk of developing IR (Insulin Resistance) and T2DM [4], while high levels of circulating insulin stimulate the ovarian theca cells to produce androgens [2].

A significant relationship between body mass index (BMI) and PCOS features, regardless of age, was demonstrated as early as 1966 based on NFBC (Northern Finland Birth Cohort) data [3].

Polycystic ovary syndrome (PCOS) is considered a chronic inflammatory condition caused (among others) by abdominal obesity or general obesity. Additionally, the inflammatory effect of nutrients, such as glucose or saturated fat promotes atherosclerotic lesions, which is emphasized in this condition [5]. In PCOS patients, glucose is found to induce oxidative stress that stimulates an inflammatory response, even in the absence of obesity. Chronic inflammation caused by poor diet in PCOS patients may form the basis of insulin resistance. Additionally, abdominal obesity in women with PCOS has been found to be involved in the development of inflammatory changes. There was a correlation between the severity of hyperandrogenism and diet-induced inflammation [5]. The influence of obesity in the course of PCOS on the increase of IR indices results in the intensification of reproductive and metabolic disorders, as well as more frequent occurrences of hirsutism, menstrual disorders, and hormonal disorders [6].

In the case of PCOS, a wide variety of balanced therapies correcting the lifestyle of patients is suggested, e.g., with the implementation of appropriate diets to minimize exposure to inflammatory factors and prevent abnormal immune system stimulation [7].

It is indicated that there is a need for further research to fully understand and support PCOS patients to implement an effective therapy taking into account dietary recommendations [1,2].

Collaboration of clinicians and specialists in many fields, including endocrinologists, cardiologists, neurologists, diabetologists, but also nutritionists, and pharmacists, can facilitate a more efficient diagnosis of the disease and the implementation of appropriate therapy, while significantly improving the quality of life of patients, but also the prognosis for this condition [1,8]. In patients with PCOS, there have been reports of noticeable deterioration in the Quality of Life (QOL), deterioration of emotional well-being and depression, and additional problems related to infertility, hirsutism, obesity, and acne. These are associated with reduced mental well-being and a lack of motivation to introduce lifestyle changes [9].

Therefore, it is believed that PCOS is an autoimmune, multigene, multifactorial, systemic, inflammatory, steroid regulation-related disease, largely associated with lifestyle errors, e.g., with inappropriate eating habits [7]. Due to the particular importance of polycystic ovary syndrome (PCOS) in the female population and due to the possibility of its severe consequences, many studies are focused on an individually adjusted diet in patients with clinically diagnosed PCOS and obesity.

## 2. Influence of Weight: Obese/Overweight PCOS Patient Groups

### 2.1. Teenagers

In the case of PCOS, it is worth paying attention to the group that includes teenagers. The first symptoms of PCOS appear in adolescence, and its pathophysiology may include the time of puberty, constituting the so-called critical development window. It is very difficult to unequivocally assess the occurrence of peri-pubertal obesity with a significant impact on the incidence of PCOS in adolescence [10]. Especially in the case of the more common forms of obesity, the clear impact on the development of PCOS is not entirely clear. However, some literature reports present analyzes and indicate that in morbid obesity before puberty with severe insulin resistance, a relationship with the development of PCOS is found [11]. A study by Laitinen et al. (2003) found an association between adolescent obesity (14 years of age) and later risk (over 60%) of developing PCOS/infertility in adult women (31 years of age). Other studies by Rich-Edwards et al. also indicated the development of infertility and ovulation dysfunction in 18-year-old girls with higher BMI [10,12,13]. It is worth paying attention to obesity in the group of teenage girls because even though it is not the only and necessary factor in the development of PCOS, it influences the occurrence and severity of this disease in adolescents. Therefore, it is suggested to analyze the possible symptoms of PCOS in obese teenage girls and the parameters of obesity, as well as comorbidities associated with it (e.g., impaired glucose tolerance, type 2 diabetes, dyslipidemia, and even depression) in adolescents diagnosed with PCOS. You can get an improvement in the relief of the reproductive and metabolic symptoms of PCOS just by losing weight [10]. The studies of Patel et al. (2017) showed that in the analyzed group of teenagers, standard lipid profiles and blood pressure were not clinically abnormal regardless of the diagnosis of PCOS. It was adolescents with PCOS that had a more atherogenic lipid profile and their arteries stiffer compared to obese adolescents without PCOS. As significant differences can be found already in comparison with the control group at the age of 15, it is worth paying attention to the sensitive, early markers of CVD risk factors in adolescents with PCOS [14]. Diet therapy and lifestyle changes can be a beneficial solution and avoid many serious health consequences at a later age.

### 2.2. Adult Women of Reproductive Age

Obesity is often one of the symptoms of PCOS in women of reproductive age. Although the complexity of the relationship between obesity and PCOS is indicated, it has been proven based on worldwide studies that it worsens the reproductive and metabolic parameters of patients with this disease [15], especially visceral obesity [16]. It is estimated that 70% of the problem of anovulatory infertility concerns patients with PCOS. In addition, the importance of weight loss and lifestyle modifications just before pregnancy is emphasized. It is indicated that weight loss improves fertility and reduces the potential adverse effects of obesity in pregnant women [17]. In a study by Welt et al. (2006) in a group of 18–45 years old patients with PCOS, irregular menstruation and hyperandrogenism, it was shown that the increase in body weight exacerbated the symptoms of PCOS (including androgen levels, ovarian volume, and insulin levels) [18]. Obesity results in an increase in insulin resistance and compensatory hyperinsulinemia as well as an increase in adipogenesis and a decrease in lipolysis. Increasing the production of androgens in the ovaries results in the enhancement of the functional hyperandrogenism of the ovaries. Additionally, as a result of obesity, there is an increase in insulin resistance and adipogenesis [16]. The results of many analyzes indicate that the metabolic syndrome may occur much more often in patients with PCOS compared to healthy people [19]. Moreover, many studies suggest that in PCOS patients it is possible to obtain improvements in the free androgen index (FAI), weight, and BMI in women with PCOS through lifestyle modifications [20]. In reproductive age patients diagnosed with PCOS, the priority over pharmacological therapy against PCOS is the prevention or treatment of obesity through intervention in lifestyle changes, as well as education and multidisciplinary care of specialists, taking into account the emotional well-being and quality of life of patients [21].

### 2.3. Adult Women over the Reproductive Age (Menopausal and Beyond)

For women with PCOS syndrome known or undiagnosed during the reproductive age, the health risks associated with PCOS extend far beyond the control of common symptoms and the treatment of infertility, as it also affects patients in the menopausal period and after its completion. Literature reports suggest long-term threats to women with PCOS as a consequence of this syndrome during and after menopause. These include, among others diabetes, obesity, dyslipidemia, hypertension, metabolic syndrome, nonalcoholic fatty liver disease, endometrial cancer, and cardiovascular disease [22]. Serum testosterone and triglyceride levels are also increased in postmenopausal women [23]. During and after menopause, the results of PCOS patients, incl. the lipid profile of women with PCOS are different from women without the condition. Inflammatory and metabolic parameters worsen with age in patients with PCOS. Hence, they still have an increased risk of developing CVD, hypertension, and type 2 diabetes. Hence, especially in elderly women with PCOS and metabolic complications, the importance of treatment including diet and lifestyle modifications is emphasized [24].

#### 2.3.1. Diet Composition

Due to the problem of obesity in PCOS, which exacerbates its clinical picture, body weight control is an important element of the therapy [21]. The analyses performed by Moran et al. (2013) comparing the effects of different diets on anthropometric, reproductive, metabolic, and psychological outcomes in women with PCOS showed promising results depending on the diet composition. Although, regardless of the composition of the diet, the use of healthy eating habits and reduced caloric intake, especially in overweight PCOS women, improve the clinical picture through weight loss [25]. There are many reports in the literature on the scope of nutritional modifications in patients with such a syndrome, incl. IR control, metabolic functions of low-calorie diets with a low proportion of simple sugars and refined carbohydrates, and the consumption of low glycemic index (GI) foods, however, there is still no unified global position on this matter [26]. Moreover, literature reports indicate the importance of lowering the proportion of saturated and trans fatty acids in the diet and the share of possible deficiencies in vitamin D, as well as chromium and omega-3 [27]. The important role of maintaining the balance of macronutrients and omega-6 and omega-3 fatty acids in the diet, which may lead to a change in the expression of inflammatory genes, is indicated [28]. It is indicated that the role of lipids in the diet influences the development of a pro-inflammatory state in the course of PCOS as its consequence [29]. The suggested diets used in patients with PCOS include slimming diets with a low content of saturated fat, with a low GI [30], as well as the Mediterranean ketogenic diet with phytoextracts (KEMEPHY) [31], and a very low-carbohydrate diet. Significant weight loss has been observed with diets rich in monounsaturated fats. Diets with a low glycemic index had a positive effect on the regulation of the menstrual cycle. With the use of high-carbohydrate diets, an increased index of free androgens was observed. In addition, the use of low-carbohydrate or low glycemic index nutrition in women with PCOS led to a marked reduction in insulin resistance, fibrinogen, total cholesterol, and high-density lipoprotein, and even improved quality of life. However, in the case of high-protein diets, an improvement in depression and self-esteem was observed [25]. The dietary intake of salt should be limited to 5 g/day, as recommended by WHO [32]. The use of spices in the diet was found to exhibit a beneficial effect on the activity of pancreatic lipase, amylase and proteases, as well as on the end digestive enzymes of the small intestine mucosa. Hence, apart from accelerated digestion, an important role of the spices used in nutrition in the reduction of the time of food transport in the gastrointestinal tract is indicated. Therefore, a significant influence of spices in the diet is suggested as stimulants of the secretion of bile in the liver necessary for the digestion and absorption of fats and stimulants of the activity of digestive enzymes [33]. Cooking methods, and even food storage conditions, have different effects on the structure and digestive properties (digestion rate, digestibility, and estimated glycemic index (eGI) value) of products, e.g., based on starch [34]. Many literature reports present a wide range of dietary options, but so far no unified position in this regard for PCOS patients. Consequently, low-calorie diets with fat modification, MD, ketogenic diets (KD), dietary GI reduction, overall reduction drive [25,35], but also additional inositol [36] are taken into account for weight loss and IR positive effects. Since so far, no worldwide, uniform guidelines on nutritional recommendations for obese PCOS patients have been developed in the case of many dietary modifications, body weight loss, improvement in BMI and parameters, including metabolism or menstrual hormonal levels, it is worth taking into account the individual needs of each patient. Diet is aimed at starting a new nutrition system, replacing the existing eating habits. In addition, during the implementation of diet therapy, every effort should be made to teach patients a rational diet and to make patients aware that diet is a way of nutrition and not a way of losing weight.

#### 2.3.2. Diets Most Applied in PCOS Treatment

In the case of overweight and obese people, it is suggested to use first-line treatment in the form of implementing appropriate eating habits in combination with lifestyle modification, followed by increasing physical activity as a second step [37]. In order to lose weight, a negative energy balance is necessary, hence the energy consumption must be consistently lower than the energy expenditure [38]. For those aiming to lose weight, not just women with PCOS, the importance of setting realistic goals is emphasized. Too restrictive and difficult to achieve assumptions often lead to failure and discouragement [39]. The goal of losing 5–10% of the initial body weight in the first six months is a reasonable and achievable assumption that results in a significant improvement in cardiometabolic risk factors [40,41]. Despite the many dietary therapies currently available that suggest promising results, it is emphasized that no single best dietary strategy for weight loss has been developed that fits patients or is used with the same effect on people suffering from the same condition [42].

### 2.4. Mediterranean Diet

The composition of the Mediterranean diet is based on the regular consumption of unsaturated fats, low GI carbohydrates, vitamins, fiber, antioxidants, and moderate consumption of animal protein [43]. It is suggested that, due to its anti-inflammatory properties and weight loss, the Mediterranean diet (MD) may have a beneficial effect in the treatment of patients with PCOS [44]. Moreover, dietary fiber in the diet is a plant material that is resistant to enzymatic digestion and includes, for example, cellulose, hemicellulose, pectic substances, and lignin. High-fiber diets (including but not limited to the Mediterranean diet) have been found to have a positive effect on health [45]. It has been suggested that increased dietary fiber consumption reduces the risk of developing, among others, coronary artery disease, hypertension, diabetes, and obesity. Increasing the consumption of this nutrient reduces blood pressure and serum cholesterol levels, and improves glycemia and insulin sensitivity. Fiber supplementation in obese people significantly supports weight loss [46]. Shai et al. compared a Mediterranean diet to a low-carbohydrate and a low-fat diet in 322 obese patients in the DIRECT study (Dietary Intervention Randomized Controlled Trial). The Mediterranean and low-carbohydrate diets did not differ from each other, but better effects of long-term weight maintenance were observed compared to the low-fat diet. In addition, it is also indicated that the introduction of Mediterranean diet foods such as extra virgin olive oil and nuts into the diet may reduce the incidence of serious cardiovascular events [42,47], which is also important from the point of view of therapy for PCOS patients. In addition, the impact of the Mediterranean diet on lowering the incidence of fasting glucose, lipids, and stroke in a population genetically susceptible to the risk of cardiovascular events is indicated (PREDIMED study; PREvención con DIeta MEDiterránea) [48]. So far, it has not been clearly established whether the individual components of the Mediterranean diet or their combination, have a beneficial effect, it has been suggested, based on epidemiological evidence, that its use brings health benefits [42]. The literature review is summarized in Table 1.

### 2.5. Low/Very Low Carbohydrate Diet

The source of the currently used low-carbohydrate diets is the formerly used low-quality Atkins ketogenic diet, based on a strong reduction in carbohydrate consumption (<30 g/d) [49]. However, the currently used low-carbohydrate diets are less restrictive and are also supported by increased consumption of fiber [42,50]. In the case of dietary fiber, the role of such components as arabinoxylan, inulin, β-glucan, pectin, bran, and resistant starches is especially emphasized. It has been shown that they contribute to the improvement of human health. Furthermore, it is suggested that the digestive and viscous properties of dietary fiber contribute to obesity and the risk of developing diabetes in the context of lowering nutrient absorption and further reducing metabolic energy and gross energy through lower energy density. In line with the published and endorsed assumption of the FDA, it is believed that a diet high in increased dietary fiber intake may reduce the incidence of coronary disease and cancer [49,50] This is important in the case of PCOS patients, especially those who are overweight and obese, where the risk of developing diabetes, coronary heart disease, and cancer may be a consequence of this syndrome.

However, clear benefits of this type of diet are seen in diabetic patients who show significant weight loss and improvement in metabolic parameters in a short time [51]. However, although there is mixed evidence, it is suggested that low-carbohydrate diets should not be used long-term due to the potential risk of increased LDL cholesterol levels and even mortality, with reduced carbohydrate intake in conjunction with excess fat intake [52]. While meta-analyzes like Nordmann et al. suggest a beneficial effect of a low-carbohydrate diet without energy limitations on triglycerides and HDL cholesterol levels [53], however, they require further research, especially in the use of long-term diet therapy, e.g., over one year [42]. An interesting solution combining the elements of the gradual introduction of the low-carbohydrate diet, and later the Mediterranean diet, which is also supported by the control of psychological parameters with modification of habits, was introduced in Greece, with promising results in obese people, under the name of Eurodieta [42]. This form of diet therapy in the context of the problems associated with PCOS syndrome, including low self-esteem and even depression faced by patients of all ages along with psychological support would provide an interesting alternative. The literature review is summarized in Table 2.

### 2.6. A Low Saturated Fat Weight-Loss Diet

Originally, very low-fat diets (10–15% total fat) were designed to prevent the development or recurrence of heart disease. The best-known programs are Ornish and Pritikin [55]. For many years, the effectiveness of low-fat diets has been emphasized, due to, among others, the fact that energy from fat is less saturating than energy from carbohydrates, the energy balance is positive and indicates weight gain in susceptible people. Based on research by Quatela et al. (2016) it is indicated that fat is more easily absorbed compared to carbohydrates [56]. Additionally, fecal energy loss is significantly lower with a high fat-to-carbohydrate ratio in the diet. Moreover, high-fat diet may cause intestinal dysbiosis by damaging the intestinal barrier, and may also adversely affect body weight and metabolic variables [42,56,57]. Therefore, the need to modify eating habits in PCOS patients is indicated, especially in the case of obesity and overweight.

However, it is indicated that very low-fat diets have a very high content of carbohydrates and fiber. Hence, the American Heart Association emphasizes that they can lead to an increase in triglyceride levels. In addition, although it may have a beneficial effect, the consumption of twice the amount of fiber recommended (40–70 g/d) may reduce the absorption of zinc, calcium, and iron [55]. Interesting observations were made in the studies of Sacks et al., in which the use of a low-fat and high-fat diet was presented. In both cases, the low consumption of saturated fat and high glycemic index (GI) foods and increased dietary fiber consumption was recommended. The studies were carried out on over 800 obese patients who received long-term (2-year) diet therapy. The diet used improved fasting lipid risk factors and insulin levels. Similar results were obtained in terms of weight loss, total and visceral fat loss, and the maintenance of lean body mass, emphasizing the importance of high standards of diet quality and the secondary contribution of macronutrient composition [42,57]. The literature review is summarized in Table 3.

### 2.7. Low Glycemic Index (GI) Diet

The first observations regarding the correlation of carbohydrate content in food products and the blood glucose concentration curve were made in the mid-1970s [59,60]. The concept of the Glycemic Index (GI) has been introduced by Jenkins et al. (1981) and representative values were tabulated [61]. The GI ranges from around 20 for fructose and whole barley to around 100 for glucose and baked potato. The physical form of the food, its processing, and the associated fat decrease the GI, and the degree of insulin response to carbohydrate-containing foods is generally compared to the glycemic response [62]. However, it is also indicated that the Glycemic Index (GI) is defined as an index relative to a component, defined as the effect of a glycemic carbohydrate in a food on blood glucose as a percentage of the effect of an equal amount of glucose [61]. It is therefore suggested that since GI is said to be static and relative, it can only be used for carbohydrate balance comparisons. In the case of diet therapy, the nutritional values must be food-based, adapted to consumption and sensitive to changes in composition. An interesting solution was proposed by Monro et al. (2003) to extend the definition of GI beyond glycemic carbohydrates and use it as a food-related indicator. Such a method could facilitate a more complete nutritional profile of the food in the design of the diet than would be the case with nutrients alone [63]. Research by Bjork et al. indicate the possibility of lowering the GI of starch products in the diet, both through the choice of raw material, but also through the optimization of its processing conditions. In addition, they suggest that some low GI foods may affect glucose tolerance at the next meal. Hence, it is concluded that some foods with a low glycemic index may be characterized by higher efficiency in modulating metabolism with prolonged use [64]. Zafar et al. review on low-GI diets and shows research conducted on people with diabetes. These diets were effective in reducing glycated hemoglobin (HbA1c), fasting glucose, BMI, total cholesterol, and LDL cholesterol, but did not affect fasting insulin, HOMA-IR, HDL, triglycerides, and insulin requirements. Particularly with long-term interventions, a marked reduction in fasting blood glucose has been observed. Hence, low GI diets are considered useful in glycemic control and weight reduction. Due to the consequences of PCOS and the metabolic parameters of patients, many authors of the studies emphasize the important role of this type of diet [65]. The literature review is summarized in Table 4.

### 2.8. KEMEPHY Diet (Ketogenic Mediterranean with Phytoextracts)

It has been suggested that ketogenic diets, including the KEMEPHY diet, are an effective and healthy way to lose weight. In addition, their use leads to an improvement in the lipid profile, a reduction in blood pressure, as well as a reduction in insulin resistance with an improvement in blood glucose and insulin levels [66], which is particularly important in the case of patients with PCOS syndrome.

The studies by Paoli et al. (2011) conducted on people with BMI ≥ 25 indicate the promising effects of the KEMEPHY diet. The introduced diet was based on green vegetables, olive oil, fish, and meat, as well as dishes composed of high-quality protein and practically zero carbohydrates, but imitating their taste, with the addition of phytoextracts. Supplementing the diet with herbal extracts was intended to reduce some of the commonly reported side effects of ketogenic diets by patients. The KEMEPHY diet uses cooked or raw green vegetables, meat, fish, eggs, and olive oil. Each meal in the studies by Paoli et al. integrated with products of high-quality proteins and maximally minimized carbohydrates. Phytoextracts are based on plant products with various effects supporting the action of the diet. Among others, mint, black radish and burdock (anti-indigestion, antioxidant and bile-enhancing properties to aid digestion), saw palmetto (regulating hormonal action), white beans (properties that inhibit alpha-amylase, supporting weight loss and glycemic control), horsetail (as an antioxidant, diuretic and helpful in glycemic control) and dandelion (as a diuretic) were used. In the analyzed group of people, there was a decrease in body weight, an improvement in cardiovascular risk indices, and a decrease in waist circumference [67]. Another 12-month study that combined the KEMEPHY diet with a traditional Mediterranean diet promoted long-term weight loss in obese subjects. Significant decreases in total cholesterol, LDLc, triglycerides, and glucose were observed. The use of the KEMEPHY diet in combination with the traditional Mediterranean diet led to an improvement in health risk factors in most of the patients studied [68]. Further studies conducted on obese PCOS patients using the KEMEPHY diet suggest promising results. Although the group of patients was not large, and the observations were carried out for 12 weeks, it can be expected that this type of diet with good cooperation with patients can bring significant effects. The KEMEPHY diet for PCOS patients may be a promising alternative to pharmacological treatment due to interesting results. This analysis showed a clear improvement in many metabolic exponents important in the case of PCOS and its complexity. Studies by Paoli et al. (2020) found a decrease in blood glucose and insulin levels and a significant improvement in HOMA-IR. Moreover, a decrease in the lipid profile markers: triglycerides, total and LDL cholesterol, and an increase in HDL levels. The LH/FSH ratio, total and free testosterone LH levels, and blood DHEAS levels were also significantly reduced. Estradiol, progesterone and SHBG levels have increased [31]. The literature review is summarized in Table 5.

### 2.9. Probiotics/Synbiotics in Diet

Obesity is a problem of a global pandemic, so it is looking for alternative methods, considering the microbiota, to apply better and better therapeutic strategies. The importance of the imbalance of the microbiome and dysbiosis, which are involved in the development of obesity and metabolic inflammation, is emphasized. Research indicates that a reduction in the microbiome’s share of beneficial microorganisms leads to dyslipidemia and the development of low-grade chronic inflammation (metabolic endotoxemia), resulting in obesity and complications in the form of comorbidities [69]. The research emphasizes the importance of dysbiosis of the intestinal microbiome in the development of metabolic disorders in patients with PCOS, due to the increase in the number of LPS-producing bacteria, with the reduction of beneficial microorganisms [2]. Numerous scientific reports have demonstrated the high therapeutic potential of probiotics/synbiotics, e.g., in obesity, insulin resistance syndrome, type 2 diabetes, and non-alcoholic fatty liver disease, and their beneficial effect of probiotics/synbiotics on the host’s health [70]. The beneficial effect of probiotics consumption by PCOS patients on the reduction of fasting blood insulin, triglycerides, and VLDL-C (very low-density lipoprotein-cholesterol) and increasing the QUICKI (quantitative insulin sensitivity check index) result is indicated [71,72]. In a comprehensive dietary intervention to prevent and treat obesity and weight loss and maintenance, the gut microbiome is an important target. It is precisely its key role in the pathogenesis of obesity and its complications. Hence, if it is balanced, a beneficial effect is observed in reducing low-grade chronic inflammation [73]. Currently, in the case of PCOS patients, the primary goal of using probiotic or synbiotic preparations (a combination of probiotic and prebiotic ingredients) in diet therapy is to correct disturbances in the intestinal microbiome. It has been suggested that gut dysbiosis accompanies obesity or may be a consequence of an unbalanced diet, and its balancing may have a beneficial effect on health through weight loss and weight maintenance [69,73]. The studies of Zarrati et al. (2014), using the probiotic bacteria L. acidophilus and B. lactis, showed a relationship between the loss of body weight and the percentage of adipose tissue [74]. In other studies, improvement of metabolic syndrome risk factors, the composition of the gut microbiome, and immune function in obese adults were found through the use of prebiotic galactooligosaccharides [75]. Supplementation with synbiotic preparations modulates the intestinal microbiome towards less proteolytic activity, positively influencing weight loss and maintenance of overweight and obese people [73,76]. When a varied diet is used, it is important to balance the gut microbiome. It has been suggested that the use of dietary therapies for weight loss based on high-protein, low-carbohydrate programs with energy restriction may be accompanied by changes in the gut microbiome associated with increased genotoxicity [77]. Research by Sergeev et al. also confirms that the use of synbiotics modulates the human gut microbiome by increasing the species diversity of microorganisms beneficial for the host, helpful in the application of a high-protein diet [73]. The role of bacteria, especially the Bifidobacterium genus, in the beneficial effects of the human microbiome and its importance in combating obesity is emphasized. Literature reports emphasize the value of using synbiotic preparations that combine strains of probiotic bacteria from the genus Bifidobacterium and Lactobacillus and galactooligosaccharides, the share of which increases after several months of therapeutic intervention [74,78,79]. In the studies by Chudzicka et al. in patients with PCOS and overweight or obesity, it was also shown that the use of synbiotic supplementation had a beneficial effect on weight loss in patients by enhancing the effect of lifestyle modification. The additional use of supplementation also led to a significant reduction in serum testosterone levels [80]. The literature review is summarized in Table 6.

The key element of diet therapy in the participants of such programs is, above all, following the recommendations of the developed eating patterns as strictly as possible. In this way, reliable information can be obtained for the interpretation of the main results [81]. The choice of diet and further nutritional behavior are influenced by many different factors, both of individual nature of patients, e.g., regarding food preferences or knowledge, support for the environment, economic factors or the availability of products. Due to the importance of efforts and goals undertaken by participants in such programs and the role of their actions in the interpretation of results, various activities are often undertaken to support the effectiveness of dietary intervention. It takes into account, among others, food delivery, close monitoring, clear dietary instructions, personalized advice, working with participants’ challenges during nutritional changes [82]. Martin C.K. et al. (2010) research also supports the assumption that constant contact between patients and a dietitian plays an important role in the effectiveness of such projects [83].

The level of satisfaction with an individually introduced diet is associated with a longer willing to participate in dietary projects. Research indicates that obese PCOS patients who stay longer in dietary intervention programs show greater weight loss and increased motivation. Due to the high involvement of patients with PCOS, their stay in the study is characterized by a different percentage of the retention rate, e.g., analyzes up to 26 weeks were achieved, among others, by 30.0% (Martin C.K, 2010). This aspect is pointed out, especially in the case of analyses with nutritional intervention in the context of long-term use. Unfortunately, the data confirm the high, as much as 77%, rate of withdrawal of participants from the program [84,85]. As additional barriers to long-term changes in lifestyle, the psychosocial aspects should also be taken into account in terms of the emotional and mental burden associated with PCOS in patients, e.g., for weight control and motivation [86]. Any type of fixed diet that has been used over a long period is not appropriate. Thus, the key element of therapy based on the use of, among others diet is precisely the education and effective cooperation of patients and specialists [87]. It is important for the duration of the study, but also for long-term changes made to eating habits and lifestyle. Women with PCOS, especially obese women, often have low self-esteem and low body acceptance. Hence, lifestyle changes related to nutrition and exercise leading to weight loss can lead to improved self-perception [88].

Faster, noticeable effects, especially in terms of weight loss, decrease in BMI and WHR (Waist-to-Hip Ratio) values, motivate patients to continue and try to make nutritional and lifestyle changes. Such opportunities may be provided to obese patients with PCOS by the use of individually adapted diets, taking into account a wide range of parameters, including low calorific value, varied protein content, and low carbohydrate content [27]. Research by Shang Y. et al. on a large group of PCOS patients confirms the significant impact of their diet to improve body composition and IR (including fasting insulin, fasting glucose, BMI, body weight and waist circumference). It is suggested that diets lowering arterial hypertension and diets with limited calories are important aspects in improving body composition in PCOS women. The longer the duration of changes in eating habits, the more favorable improvement in parameters is observed, comparable even to pharmacological treatment with metformin in PCOS patients [89].

The studies by Phy J.L et al. (2015), conducted in obese and overweight PCOS patients on an 8-week insulin-free diet, showed a marked improvement in many parameters. They showed an improvement in anthropometric parameters, a decrease in body weight, a decrease in waist circumference and adipose tissue as well as an improvement in insulin sensitivity according to HOMA-IR, as well as a decrease in total and free testosterone. The studies also emphasized that the patients did not have to count calories and carbohydrates or take medications during this dietary intervention [90]. Although the results of such a study are promising, the duration of the therapeutic intervention is quite short to unequivocally assess its impact on the disease of PCOS.

The global analyses emphasize the relationship between obesity in patients with PCOS and the occurrence of dysbiosis in the intestinal microbiome [2,91,92]. In women using synbiotic supplementation, the “healthier” intestinal microbiome was restored. The exposure of young women to weight gain and the difficulties associated with achieving an appropriate weight was also reported as important factor [93,94]. Moreover, the consequences of obesity in women, especially young ones, include the development of several disorders, not only of a metabolic and circulatory nature, but also neoplasms, incl. colon, breast, uterus and, just with the development of PCOS, infertility and pregnancy complications [95,96]. It is also emphasized that the prevalence of overweight and obesity in women with PCOS, which result in hypertrophy or hyperplasia of adipocytes, is important. As a result of adipocyte hypertrophy, a hypoxic microenvironment with the proinflammatory secretion of cytokines, “flooding” of free fatty acids, and invasion of macrophages and IR occurs [97]. In obese women with PCOS, especially as a result of the development of insulin resistance (IR), there is an increase in the concentration of free fatty acids and triglycerides in the serum, which leads to an increase in de novo hepatic lipogenesis and hyperlipidemia [98], and increased fat storage in skeletal muscle, liver, and pancreas [99]. Many studies emphasize the importance of obesity in the development of inflammation in metabolic tissues. Metabolic cells, in response to excess nutrients and energy, have been indicated to direct a process defined as low-grade chronic inflammation known as metaflammation [100].

The effect of synbiotics, especially with the participation of Bifidobacterium spp., on the improvement of weight loss parameters, BMI, WHR values, and the potential influence of, among others, on the lipid profile confirm the importance of the gut microbiome. Research by Collado et al. (2010) reports that bifidobacterial dysbiosis precedes obesity [101], and is associated with body weight, weight gain, and metabolic biomarkers [102]. Moreover, an abnormal number or composition of Bifidobacterium spp. is probably the most frequently observed change in the gut microbiome, which occurs in many different diseases. Moreover, an important role has been suggested, that *Bifidobacterium* spp. play in the maintenance of intestinal homeostasis and may be a potential biomarker of intestinal health and the development of dysbiosis. Moreover, the increase in the share of bifidobacteria in the gastrointestinal tract may be prophylactic and/or alleviate the symptoms of diseases related to the influence of microbiota disorders. The use of probiotics or synbiotics in therapies, especially in obese individuals, may form the basis of a dietary intervention strategy to improve or alleviate intestinal dysbiosis [103]. Synbiotics combine strains of probiotic bacteria from the genus Bifidobacterium and Lactobacillus with incl. fructooligosaccharides. Fructooligosaccharides (FOS) are recognized as having a beneficial effect on the health of the host by promoting the growth of beneficial bacteria such as the *Bifidobacterium* population (Mikkelsen and Jensen, 2004), and have a beneficial effect on the possibility of bacterial colonization in a competitive gut ecosystem [104]. In addition, studies on mice by Benyacoub et al. suggest that a diet with the addition of FOS and inulin stimulates the immunity of the mucous membranes [105,106]. Additionally, in the studies by Ahmadi et al. During the 12-week probiotic supplementation in a group of 60 PCOS patients and in the analyses with synbiotics by Samimi et al., a beneficial effect on weight loss, insulin resistance markers, triglycerides and VLDL cholesterol was shown [72,107].

## 3. Conclusions

Since the current therapies used in women of all ages with PCOS do not provide the possibility of a full recovery, apart from using drugs lowering the concentration of insulin and hormonal drugs, alternative methods are sought, considering changes in lifestyle and diet. In the professional care of patients with PCOS, the key role of doctors is emphasized, among other gynecologists and endocrinologists, but also nutrition specialists. The joint and comprehensive action of specialists in many fields is to help in achieving the goals of improving the parameters of the lipid profile, weight, and BMI, but also in making informed and long-term decisions by the PCOS patients in terms of a healthy lifestyle [108]. In obese patients with PCOS motivational support, control, and monitoring of implemented recommendations are crucial. Regardless of the selection of the so-called diet therapy, sometimes supported by increased physical activity, the overriding goal is to maintain a healthy body weight or reduce it, especially in the case of high BMI [87]. An important role in the treatment of obese women with PCOS is played by introducing lifestyle changes, especially in terms of nutritional changes, and striving to reduce their body weight by at least 5–10% [26]. The inclusion of synbiotic supplementation in the therapy of women with PCOS, apart from changing dietary habits, serve as a starting point for further long-term analyzes. Assessment of the impact of therapy enriched, especially with synbiotics, on the improvement of, among others, metabolic parameters is a very promising solution, but it certainly requires long-term analyzes and further observations.

## Figures and Tables

**Table 1:** Effects of a Mediterranean Diet (MD).

Author	Year & Ref	Assumptions/Methodology		Results
Barrea et al.	2019 [44]	112 patients with PCOS and 112 controls	Use of Mediterranean Diet (MD) and nutritional pattern compliance assessment based on PREvención con DIetaMEDiterránea (PREDIMED) and 7-day dietary records.	A direct relationship has been shown between MD adherence and clinical severity of disease in women with PCOS, possibly including inflammation, IR, and hyperandrogenemia.
Corella et al.	2013 [48]	Randomized study (2 MedDiet intervention groups and a control group). 7018 participants.	The PREvención con DIetaMEDiterránea (PREDIMED) study. Assessment of major cardiovascular events. Data were analyzed at baseline and after a median follow-up of 4.8 years.	MedDiet reduces the rise in fasting glucose and lipids and reduces the incidence of strokes.
Anderson et al.	2009 [46]	Analysis based on epidemiological studies reporting dietary fiber consumption, assessed in 1993–2000 (children and adults)	Dietary fiber intake	High fiber intake is protective and beneficial in treating the disease and, for example, a lower risk of developing CHD, stroke, high blood pressure, diabetes, obesity. It improves serum lipoprotein values, lowers blood pressure, improves blood glucose control in people with diabetes, and supports weight loss. Inulin and some soluble fibers enhance immune function.
Shai et al.	2008 [47]	2-year study of 322 moderately obese subjects	Diet intervention included MD	Beneficial effect of MD on glycemic control.
Koliaki et al.	2018 [42]		Review of general principles and recommendations for obesity dietary management and elements of optimal nutritional intervention	The effectiveness of MD in slimming is indicated similar to low-carbohydrate diets, however, thanks to the balanced composition and variety of pro-health micronutrients, the potential benefits for overall health are emphasized

**Table 2:** Effects of Low/Very Low carbohydrate diet.

Author	Year & Ref	Assumptions/Methodology	Results
Koliaki et al.	2018 [42]	Review of general principles and recommendations for obesity dietary management and elements of optimal nutritional intervention	A low-carbohydrate diet can be effective and metabolically beneficial in the short term, and long-term compliance differs, depending on the nutrient content, patient health, and risk factor profile.
Astrup et al.	2004 [54]	A systematic review of low-carbohydrate diets including long term randomized trials in obese patients.	Weight loss has been shown to be better on a low-carbohydrate diet after 6 months, and no difference after 12 months of diet therapy. The relationship between the weight loss achieved and the duration of the diet and the restriction of energy consumption was emphasized, not directly with the restriction of carbohydrates.
Bueno et al.	2013 [51]	Metanalysis with thirteen randomized controlled trials, including VLCKD (very-low-carbohydrate ketogenic diets) in diet therapy.	It was found in the long term that VLCKD was more effective in achieving greater weight loss compared to people who used LFD (low fat diet), suggesting that such a diet is more effective in the fight against obesity.
Noto et al.	2013 [52]	Metanalysis based on 17 studies with a group of 272,216 people and using a low-carbohydrate scale. To analyze the long-term impact of such a diet (low carbohydrate diet sometimes in combination with high protein diet) on mortality and CVD (cardiovascular disease) incidence.	Data showed that a higher risk of death from any cause was associated with low-carbohydrate diets, but they were not significantly associated with the risk of mortality and CVD morbidity.
Nordmann et al.	2006 [53]	Metanalysis based on studies that showed changes in body weight by intention-to-treat analysis with a minimum follow-up of 6 months. The analysis included five studies and 447 people in total.	Diets without energy restriction have been found to be comparable to energy-reduced low-fat diets in achieving weight loss for up to 1 year. Favorable changes in triglyceride and cholesterol values in high-density lipoproteins and potential unfavorable changes in cholesterol values in low-density lipoproteins were indicated.

**Table 3:** Effects of Low saturated fat weight-loss diet.

Author	Year & Ref	Assumptions/Methodology		Results
Koliaki et al.	2018 [42]	Review of general principles and recommendations for obesity dietary management and elements of optimal nutritional intervention		Compared to regular diets, low-fat diets are more effective at reducing weight with little to moderate effect. However, compared to high-fat diets such as low-carbohydrate diets, low-fat diets are equally or less effective at achieving significant long-term weight control.
Quatela et al.	2016 [56]	Systematic analysis of the effects of varied energy intake, macronutrient composition, and the nutritional pattern of meals consumed after an overnight fast on diet-induced thermogenesis (DIT).		It has been shown that the amount of DIT increase is influenced by energy consumption, macronutrient composition and diet.
Green et al.	1997 [58]	Eighteen normal-weight young male University students	Subjects ate a low-energy (2238 kJ) or high-energy (3962 kJ) lunch, and 2 h later were free to consume any high fat, low sucrose or high sucrose low fat snacks.	Low-energy lunch resulted in higher hunger levels and higher consumption (*p* < 0.01). The results indicate that the size of the eating episode depends on the level of hunger and the nutritional composition of the food consumed.
Sacks et al.	2009 [57]	A group of 811 overweight adults were allocated to one of four diets	The target fat, protein and carbohydrate energy percentages were 20, 15 and 65%; 20, 25 and 55%; 40, 15 and 45%; and 40, 25 and 35%. The diets consisted of similar foods and met cardiovascular health guidelines. Training sessions were offered to participants for 2 years.	The primary outcome was the change in body weight after 2 years in a factorial comparison of low fat versus high fat and mean protein versus high protein content twice, and a comparison of highest and lowest carbohydrate content. Similar results in weight loss were shown for each of the diets used.

**Table 4:** Effects of Low glycemic index (GI) diet.

Author	Year & Ref	Assumptions/Methodology		Results
Fujita et al.	1975 [60]	22 volunteers, 20–40 years old, with no personal or family history of diabetes, weighing 61 to 105 kg.	3 dietary regimens were used: low-carbohydrate diet (12 gm. carbohydrate, 260 g. proteins, 190 gm. fat and 2870 calories); a composed moderately high carbohydrate diet (390 gm of carbohydrates, 36 gm of protein, 120 gm. fat and 2784 calories); and a very high-carbohydrate diet (composed of 5–10 gm of carbohydrates, 72 g fat, 44 gm. proteins and 2843 calories).	There was a clear effect of carbohydrate consumption on insulin and glucagon responses to a protein meal. After a week of carbohydrate restriction, insulin increased less than glucagon.
Truswell et al.	1992 [62]	Analysis of GI methodology and food factors influencing the glycemic response.		Physical food form, processing and fat content have an effect on GI, reduce GI and be associated with delayed gastric emptying. It was emphasized that the degree of insulin response to carbohydrate-containing foods is similar to the glycemic response and the predictability of the GI of a composite meal. The importance of the concept of the gastrointestinal tract in designing a diet in the prevention and treatment of diabetes, especially of the non-insulin-dependent type was emphasized. In addition, in determining satiety and the impact of low GI products on aging.
Monro et al.	2003 [63]	Analysis of the effectiveness of the application and the possibility of redefining the assumptions and limitations of the glycemic index in the dietary management of postprandial glycemia		The analysis suggests extending the GI definition and using it as a food related index, expressed as glycemic glucose equivalent (GGE)/100 g food and extrapolated to GGE according to a common standard measure (CSM) as the glycemic load value of known amounts of food.
Bjorck et al.	2000 [64]	Research analysis of GI reduction of the selected starch products and/or optimization of their processing conditions.		It has been suggested that some low GI foods may be more effective in modulating metabolism in the long run.
Zafar et al.	2019 [65]	Meta-analysis based on 54 randomized controlled trials in adults or children with impaired glucose tolerance, type 1 diabetes or type 2 diabetes.	Low GI diets	Low GI diet have been shown to be effective in reducing glycosylated hemoglobin (HbA1c), fasting glucose, BMI, total cholesterol, and LDL cholesterol, but there is no effect on fasting insulin, HOMA-IR, HDL, triglycerides, and insulin requirements. Decreases in fasting glucose and HbA1c were inversely correlated with body weight. In the studies with the longest duration, the greatest decreases in fasting blood glucose were observed. Low GI diets are believed to be helpful in glycemic control and weight reduction in people with prediabetes or diabetes.

**Table 5:** Effects of KEMEPHY diet.

Author	Year & Ref	Assumptions/Methodology		Results
Perez-Guisado et al.	2008 [66]	Prospective study in 31 obese subjects (22 men and 19 women), body mass index and age 36.46 ± 2.22 and 38.48 ± 2.27, respectively.	Caloric unlimited ketogenic Diet—“Spanish Ketogenic Mediterranean Diet” (SKMD) with the inclusion of extra virgin olive oil as the main source of fat (≥30 mL/day), moderate consumption of red wine (200–400 mL/day), green vegetables and salads as the main source of carbohydrates and fish as the main source of protein.	There was a significant (*p* < 0.0001) reduction in body weight, body mass index, systolic blood pressure, diastolic pressure, total cholesterol, triacylglycerol and glucose levels. There was a significant (*p* = 0.0167) reduction in LDLc and an extremely significant increase in HDLc. The efficacy and safety of SKMD in weight loss was demonstrated, promoting non-atherogenic lipid profiles, lowering blood pressure and improving fasting blood glucose levels.
Paoli et al.	2011 [67]	The study group consisted of 106 people with a body mass index ≥ 25, aged 18 to 65 years (19 men and 87 women; mean age 48.49 ± 10.3).	Modified ketogenic diet based on green vegetables, olive oil, fish and meat, and meals consisting of high-quality protein and virtually zero carbohydrates, with the addition of herbal extracts (KEMEPHY ketogenic Mediterranean with fitoextracts). The calories in the diet were unlimited.	There was a significant (*p* < 0.0001) decrease in BMI, body weight, percentage of adipose tissue mass, waist circumference, total cholesterol, LDLc, triglycerides and blood glucose. There was a significant (*p* < 0.0001) increase in HDLc. It was observed that following the KEMEPHY diet resulted in weight loss, improved cardiovascular risk indices and a reduction in waist circumference.
Paoli et al.	2013 [68]	89 male and female obese subjects, aged between 25 and 65 years who were overall healthy apart from being overweight.	12-month diet protocol: 20 days of KEMEPHY; 20 days of low carbohydrate non-petogenic; 4 months Mediterranean normocaloric nutrition; a second 20-day ketogenic phase followed by 6 months of Mediterranean normocaloric nutrition.	In majority of patients (88%) there was significant loss of body weight, BMI, and body fat during both ketogenic phases followed by successful maintenance.
Paoli et al.	2020 [31]	Fourteen overweight women with diagnosis of PCOS.	Ketogenic Mediterranean diet with phyoextracts (KEMEPHY) for 12 weeks.	After 12 weeks, there was a significant reduction in body weight, BMI, FBM—fat body mass and VAT (visceral adipose tissue). In addition, a significant, slight decrease in LBM (lean body mass), a decrease in blood glucose and insulin levels, and a significant improvement in HOMA-IR. There was a significant decrease in triglycerides, total cholesterol, and LDL cholesterol with increasing HDL levels. The LH/FSH ratio, the level of total and free testosterone LH and the level of DHEAS in the blood were lowered. Increase in estradiol, progesterone and SHBG (sex hormone binding globulin).

**Table 6:** Effects of probiotics/synbiotics in diet.

Author	Year & Ref	Assumptions/Methodology		Results
Liu et al.	2017 [2]	The study included 33 PCOS patients (non-obese PCOS patients, PN, n = 12; PCOS obese patients, PO, n = 21) and 15 controls (non-obese controls, CN, n = 9; obese control group, CO, n = 6).	All subjects were tested in the morning after an overnight fast (≥8 h). Clinical characteristics and metabolic profiles were determined. Anthropometric parameters, blood RT, sex hormones, metabolic parameters, mediators of the brain-gut axis and psychological scales were analyzed. Blood (e.g., testosterone, ghrelin, serotonin) and stool (Sequencing of the V3–V4 region of the 16S rRNA gene for gut microbiome) tests were performed.	In women with PCOS, intestinal bacterial dysbiosis has been shown to be associated with disease phenotypes.
Markowiak et al.	2017 [70]	Analysis of the research on the impact of probiotics, prebiotics and synbiotics on human health and their beneficial effects and effectiveness in human nutrition.		The key role of probiotic microorganisms in maintaining the balance of the human intestinal microbiome was emphasized, as well as the high therapeutic potential, e.g., in obesity, insulin resistance syndrome, type 2 diabetes and non-alcoholic fatty liver disease.
Liao et al.	2018 [71]	Meta-analysis of randomized controlled trials (RCTs) in women with PCOS to evaluate the effects of probiotic supplementation on glycemic control, lipid profiles, weight loss, and C-reactive protein (CRP). 26 publications were screened and 6 RCTs involving 406 PCOS participants (aged 25–28.5 years).		In patients with PCOS, the beneficial effect of daily consumption of probiotics on the reduction of FBI (fasting blood insulin), Triglicerydes and VLDL-C (very low density lipoprotein-cholesterol) and an increase in the QUICKI score was shown.
Ahmadi et al.	2017 [72]	Randomized, double-blind, placebo-controlled study in 60 women with PCOS.	Probiotic probiotic (n = 30) or placebo (n = 30) supplementation for 12 weeks.	12-week probiotic supplementation from PCOS women has been shown to have beneficial effects on weight loss, insulin resistance markers, triglycerides, and VLDL cholesterol levels.
Sergeev et al.	2020 [73]	A group of 20 weight loss participants, male and female, who were randomly assigned to placebo (control) or synbiotics (treatment). The participants were initially overweight/obese and had an average BMI of 33.5 kg/m^2^.	The placebo group (n = 10) followed a weight loss program (low-carbohydrate and high-protein diet with reduced energy pattern). The synbiotic group (n = 10) followed the same nutritional plan but additionally received a synbiotic supplement (probiotic plus prebiotic) daily for 3 months. The control group received a placebo supplement with a similar appearance and the same energy content as the synbiotic supplement.	Based on the results, it was found that the synbiotic supplementation modulates the human gut microbiota by increasing the abundance of potentially beneficial microbial species.
Zarrati et al.	2014 [74]	A randomized, double-blind clinical trial in 75 healthy overweight and obese subjects.	Study participants were randomly assigned to eat plain yogurt on a low-calorie diet (n = 25) or receive probiotic yogurt with LCD (n = 25) or consume probiotic yogurt without LCD (n = 25) for 8 weeks.	The weight loss diet and probiotic yogurt had a synergistic effect on the expression of T-cell genes in PBMC (peripheral blood mononuclear cells), fat percentage, and body weight in overweight and obese people.
Vulevic et al.	2013 [75]	45 overweight adults with ≥3 risk factors for metabolic syndrome in a double-blind, randomized placebo (maltodextrin) group, cross-over study (with a 4-week washout period between interventions).	A mixture of galactooligosaccharides [Bi2muno (B-GOS)]. Pełna krew, ślina, kał i pomiary antropometryczne zostały wykonane na początku, 6 tygodniu i końcu każdego 12-tygodniowego okresu interwencji.	The administration of B-GOS had a beneficial effect on the composition of the intestinal microbiome, the immune response and the concentration of insulin, TC and TG.
Ferrarese et al.	2018 [76]	Analysis of trials with probiotics/prebiotics and synbiotics in the fight against obesity/weight loss/metabolic syndrome and clinical trials and relevant preclinical outcomes based on molecular mechanisms.		It has been shown in large independent studies that dietary supplementation with synbiotics (e.g., with Lactobacillus gasseri) has been shown to reduce weight and have anti-inflammatory effects. The addition of galactomannan and/or inulin fibers may enhance the effects of weight control.
Seganfredo et al.	2017 [78]	A systematic review based on 43 studies to evaluate the relationship between the gut microbiota and weight loss in overweight/obese adults and its potential modification in the treatment of obesity.		Based on the analysis of the results, it was shown that restrictive diets and bariatric surgery disrupt the microbiome and have the potential to lead to long-term harmful changes in the colon. In contrast, prebiotics can restore a healthy microbiome and reduce body fat.
Chudzicka-Strugała et al.	2021 [80]	65 women diagnosed with PCOS and body mass index (BMI) > 25. The patients were divided into 2 groups: placebo and synbiotic. Both groups had identical lifestyle modifications (closely monitored diet and exercise regimen). Diet—limiting caloric intake from 1400 to 1800 kcal/day based on body composition analysis and alcohol exclusion. Exercise—walking every day for 30 to 40 min. The placebo group received placebo capsules and the synbiotic group received a synbiotic supplement.	A randomized (1:1) double-blind, placebo-controlled trial. Assessments were made at the beginning and repeated after 3 months of treatment. Lifestyle modifications in combination with synbiotic or placebo supplementation.	Supplementation with synbiotics intensified the effect of lifestyle modification on weight loss and led to a significant decrease in serum testosterone levels.

## Data Availability

Not applicable.
